# A smartphone-based crowd-sourced real-time surveillance platform (apple snail inspector) for the invasive snails: a design and development study

**DOI:** 10.1186/s13071-024-06182-z

**Published:** 2024-02-21

**Authors:** Qiang Zhang, Xin Ding, Yingshu Zhang, Yougui Yang, Fanzhen Mao, Bixian Ni, Yaobao Liu, Richard Culleton, Yang Dai, Jun Cao

**Affiliations:** 1 National Health Commission Key Laboratory of Parasitic Disease Control and Prevention, Jiangsu Provincial Key Laboratory on Parasite and Vector Control Technology, Jiangsu Provincial Medical Key Laboratory, Jiangsu Institute of Parasitic Diseases, Wuxi, Jiangsu China; 2 Center for Global Health, School of Public Health, Nanjing Medical University, Nanjing, Jiangsu China; 3 Division of Molecular Parasitology, Proteo-Science Centre, Ehime University, Matsuyama, Ehime Japan

**Keywords:** Apple snails, Mobile health, WeChat, Real-time surveillance, Design and development

## Abstract

**Background:**

The large amphibious freshwater apple snail is an important invasive species in China, but there is currently no method available for their surveillance. The development and popularization of smartphones provide a new platform for research on surveillance technologies for the early detection and effective control of invasive species.

**Methods:**

The ASI surveillance system was developed based on the infrastructure of the WeChat platform and Amap. The user can directly enter the game interface through the WeChat port on their mobile phone, and the system automatically obtains their location. The user can then report the location of apple snails. The administrator can audit the reported information, and all information can be exported to Microsoft Excel version 2016 for analysis. The map was generated by ArcGIS 10.2 and was used to characterize the spatial and temporal distribution of apple snails in Jiangsu Province.

**Results:**

The architecture of ASI consists of three parts: a mobile terminal, a server terminal and a desktop terminal. We published more than 10 tweets on the official WeChat account of the system to announce it to the public, and a total of 207 users in 2020 and 2021 correctly reported sightings of apple snails. We identified 550 apple snails breeding sites in 2020 and 2021, featuring ponds (81%), parks (17%) and farmland (2%). In addition, most of the locations contained snail eggs, and the reporting times mainly occurred between May and September.

**Conclusions:**

The ASI is an effective surveillance system that can be used to identify the breeding locations of apple snails and provides the basis of prevention and control for its dispersal. Its successful development and operation provide new potential avenues for surveillance of other public health issues.

**Graphical Abstract:**

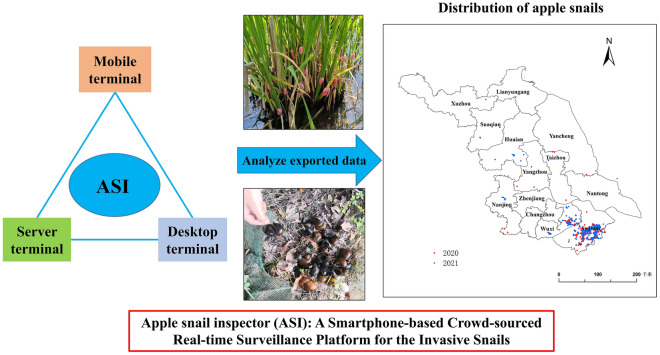

**Supplementary Information:**

The online version contains supplementary material available at 10.1186/s13071-024-06182-z.

## Background

The apple snail from the genus *Pomacea* (Caenogastropoda: Ampullaridae) is an invasive pest that is listed among “100 of the world’s worst invasive species” [1]. It does great harm to aquatic crops and is considered a major dangerous and invasive organism affecting agriculture by the State Environmental Protection Administration of China [2]. In addition to the risk of injury to farmers stepping on their sharp shells, the large amount of feces they produce pollutes fresh water bodies [3]. Furthermore, they compete with local species for resources, leading to endangerment or even local extinction of freshwater organisms, seriously affecting the biodiversity of these habitats [4–6]. Furthermore, they can also act as the intermediate host of pathogens such as the parasitic nematode *Angiostrongylus cantonensis*, which infects humans through the ingestion of raw or undercooked mollusks carrying infective larvae and causes eosinophilic meningoencephalitis and even death in case of severe infection [7, 8]. These snails were first introduced to Taiwan (Province of China) from Argentina as a high-protein economic species and were then bred in Guangxi, Fujian, Zhejiang and Jiangsu provinces [9, 10]. Due to their high reproductive output, low market economic benefits and poor taste, apple snails were subsequently abandoned in the wild and rapidly formed a natural population in many southern areas of China owing to their adaptability, rapid growth and short reproductive cycle [11].

Jiangsu, a coastal province in eastern China, has a rich water network that is suitable for the growth and reproduction of apple snails. Previous studies have shown that apple snails have caused significant economic losses to agriculture and aquaculture in the province [12]. They have also had a serious impact on urban biodiversity and the natural environment in many areas and now constitute a risk to the natural ecology and poses a biosafety problem [13]. Therefore, detailed information on the distribution of apple snails is needed to prevent their continued diffusion and to facilitate their control. No surveillance system for this invasive species of snails is currently available, and information on the scale of apple snail invasion is lacking. The snails often lay their eggs on the bases of buildings, about 20 cm above the surface of the water or on the stone walls of river banks. The eggs are bright pink-red in color so can be easily observed by non-experts. This offers promise for crowd-sourced surveillance of apple snails.

Traditional disease surveillance systems have certain shortcomings, including lagging of reports and outdated reporting, incomplete coverage and being labor intensive. These difficulties have led to the development of novel surveillance systems such as internet-based disease surveillance systems [13, 14]. Mobile phones have provided the global health community with innovative and cost-effective strategies to address many challenges in healthcare [15, 16]. Mobile health (mHealth) is a concept that involves the use of mobile communication devices, such as mobile phones, to deliver services through apps, SMS, etc., and has the advantages of low cost, convenient management, simple operation and the ability to locate users through global positioning systems (GPS) [15]. mHealth technology has been used as a surveillance system that can satisfy the demands for real-time disease surveillance and identify the emergence of epidemics [17]. Several mobile applications (apps) have been developed for the surveillance of epidemics, including those for reporting and mapping cases of influenza, H1N1, Ebola, Zika and dengue [18, 19]. Most of these apps are free and can provide real-time tracking as well as interactive maps [20, 21]. Since 2000, *A. cantonensis* has been detected and caused outbreaks in many places in China [22–25], posing a serious threat to human health and attracting widespread attention from the whole society. Given its large environmental and public health importance, the development of mHealth-based technology for the surveillance of apple snails is highly desirable.

WeChat, a free application released in 2011 by Tencent, Inc., is the most widely used social networking platform in China. It had over 1.2 billion active monthly customers from 200 countries communicating in 20 languages in the third quarter of 2020 [26]. Like other social media platforms, WeChat allows users and entities to create accounts. These public accounts allow corporations, news agencies and government departments to distribute information through text, video and audio, including public health-related updates [27]. A variety of health-related information is continually generated and transmitted among users through the different functions of the platform. This makes WeChat promising for disseminating general information on public health [28].

We developed an mHealth-based surveillance system, named the “Apple Snail Inspector” (ASI), to provide information on the distribution of apple snails in Jiangsu Province using the WeChat platform. The system allows the public to take photographs of apple snails (eggs or snails) and upload them via mobile phone at any time where there is a cellular signal while sharing their location. The system improves public awareness of the hazards posed by apple snails and can be used to efficiently locate them. This provides a basis for the prevention and control of this invasive species.

## Methods

### Development and design of the ASI system

JavaScript code and HTML5 syntax were used to develop the ASI system. It is based on the infrastructure of the WeChat platform and Amap (Amap is China’s leading provider of digital map content, navigation and location service solutions. It has Class A surveying and mapping qualifications for electronic navigation maps and internet map services). The overall architecture consists of three parts: a mobile terminal, a server terminal and a desktop terminal. The mobile terminal is responsible for field photography, data collection and positioning. The map is based on the Amap engine and enables drawing and editing at the mobile terminal. The server terminal mainly provides infrastructure and standard services and is responsible for uploading the data and photographs collected by the mobile terminal. The main functions of the desktop terminal include loading data packages, graphics editing and generating the relevant charts.

The system consists of four layers: infrastructure, data, application support and application. They are designed in accordance with the software specifications. The infrastructure layer is composed of hardware control equipment related to the main functions, including photography and GPS services. The application support layer is responsible for GIS services, data interaction and data services. The application layer is responsible for data organization and management, basic logic control and function customization. Each layer is developed and designed according to the principle of mutual non-influence whereby the layers operate independently of one another to facilitate the maintenance of and subsequent improvements to the software.

### Data management and verification of background information

When users find potential apple snails and eggs, they can take photos of them and upload them to the ASI mobile client. The system automatically acquires the geographical location of the photograph. The reported information can then be verified at the computer management terminal, and correctly reported information is rewarded (1 to 20 CNY at random). The reward is similar to a form of game, which can greatly increase the enthusiasm of participants. Finally, the system characterizes the temporal and spatial distributions as well as the abundance of apple snails and exports the data to a Microsoft Excel file for analysis. Determining the accuracy of the reported information is an important means of obtaining the distribution of apple snails in the province. (i) The reported photos need to have been taken on site, not online. (For example, some users take some photos downloaded from internet directly for reporting rather than live photos). (ii) The location and time reported need to be reasonable. (For example, some reported addresses display indoors locations, where it is impossible for snails to breed. In addition, some photos show daytime while the reported time shows nighttime, which is illogical). (iii) The pictures need to feature the snails or their eggs. Only if these three criteria were met did we verify results and release the rewards.

### Promotions of the surveillance system

People frequently go outdoors on holidays (e.g. the Dragon Boat Festival and May Day) and weekends. We used tweets on WeChat to promote the ASI app. They provided detailed information on apple snails and an introduction to operating our surveillance system. We shared the tweets with our circle of friends on WeChat and asked the county-level and municipal-level centers for disease control and prevention of Jiangsu Province to publicize our surveillance system to cover the entire province as much as possible.

### Ethical considerations

This study was approved by the Ethics Review Committee of Jiangsu Institute of Parasite Diseases. All the reported information was used only for scientific purposes and the study data were anonymized and de-identified. All forms containing personal information were coded and stored in a password-protected database and computer.

### Statistical and data analysis

The data were exported to Microsoft Excel (version 2016) for analysis. The map was generated by ArcGIS 10.2. We used Chi-square test to analyze the differences in the distribution of confirmed breeding sites reported in 2000 and 2021 among users, with the significance level set to *P* < 0.05. Statistical analyses were performed using SPSS, version 23.

## Results

### Introduction to the operation of mobile surveillance system ASI

**Fig. 1:**
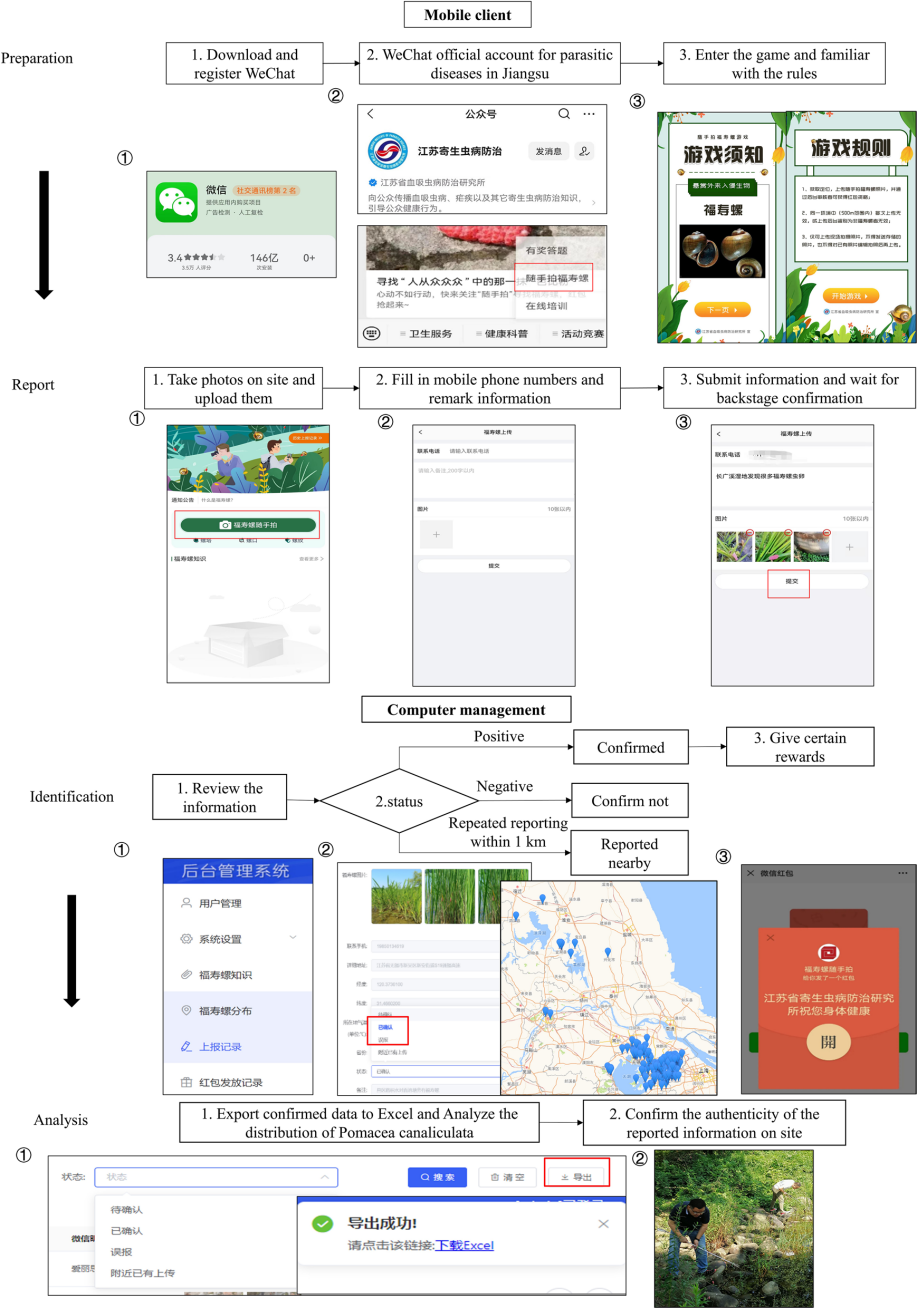
Introduction to the operation of the ASI and the corresponding screenshots of its mobile client and computer terminal

The ASI is a non-commercial system that allows for the real-time reporting and mapping of the distribution of apple snails. As shown in Fig. 1, the operation of the system is divided into those of the mobile client and those of the computer terminal. It consists of several processes, including preparation, reporting, identification and analysis.

The upper part of Fig. 1 shows the layout of the screen of the mobile client. In the preparation stage, users need to download and register WeChat and follow the official WeChat account of the Jiangsu parasitic disease control. The user can then click the ASI button to enter the game. The game interface provides users with general information on apple snails and the rules of the game. In the reporting stage, the user needs to take photos on site, and the system automatically searches for the reported point by using the location of their mobile phone. The user also needs to provide their mobile phone number and certain information for subsequent on-site verification. After completing these operations, the user can submit information and wait for verification of the background. The bottom half of Fig. 1 shows the layout of the screen of the computer management terminal. In the identification stage, the manager reviews the uploaded information. They need to choose the status of the report according to the relevant photos and locations uploaded by the user. If the photos are confirmed to be those of apple snails or their eggs, and their location is being reported for the first time, the manager clicks the “Confirmed” button and the background management automatically sends certain rewards to the users. For all correctly reported locations, the coordinates are automatically generated on the map to allow us to clearly see the distribution of apple snails. To avoid redundant reporting, we set the distance between the reporting locations to > 1 km; otherwise, we chose the “Reported nearby” button. If the reported results are reviewed and found to be incorrect, their status is “Not confirmed.” In the analysis stage, all results are exported to Microsoft Excel. We can then analyze the correct results to clarify the distribution of apple snails in the province. As a lot of information was reported, we randomly selected some reported points distributed in different parts of our province for on-site confirmation, and the results showed that we could indeed find the snails on site, which was consistent with the reported information.

### Operating scenarios of the ASI surveillance system

The ASI system was designed and developed in 2020. We worked on its functioning, ease of use, navigation, flow, logic and aesthetics before officially putting it into operation. We invited colleagues and professional technicians to conduct a simple internal test of it, and all testers praised the system. We published more than 10 tweets on the official WeChat account of the system to announce it to the public (Additional file 2: Fig. S1), and thousands of people eventually participated in our game. As shown in Table 1, a total of 207 users in 2020 and 2021 correctly reported sightings of apple snails, and most users successfully reported one to three locations. There was no statistically significant difference in the distribution of the numbers of reported confirmed breeding locations in these 2 years (Chi-square test, *χ*^2^ = 0.108, *df* = 2, *P* = 0.948). To thank these users for their successful reporting, we have thus far sent 550 rewards worth > 5000 CNY. Participation in the game by the public continues to increase, and users have reviewed our system positively.

**Table 1:** Distribution of the numbers of reported confirmed breeding locations by users in 2000 and 2021

Numbers of reported confirmed breeding locations	Number of users		Total	Statistical analysis
	2020	2021		
1–3	82	98	180	*χ*^2^ = 0.108, *df* = 2, *P* = 0.948
4–6	9	12	21	
≥ 7	3	3	6	
Total	94	113	207	

### Two-year reporting of apple snails

**Fig. 2:**
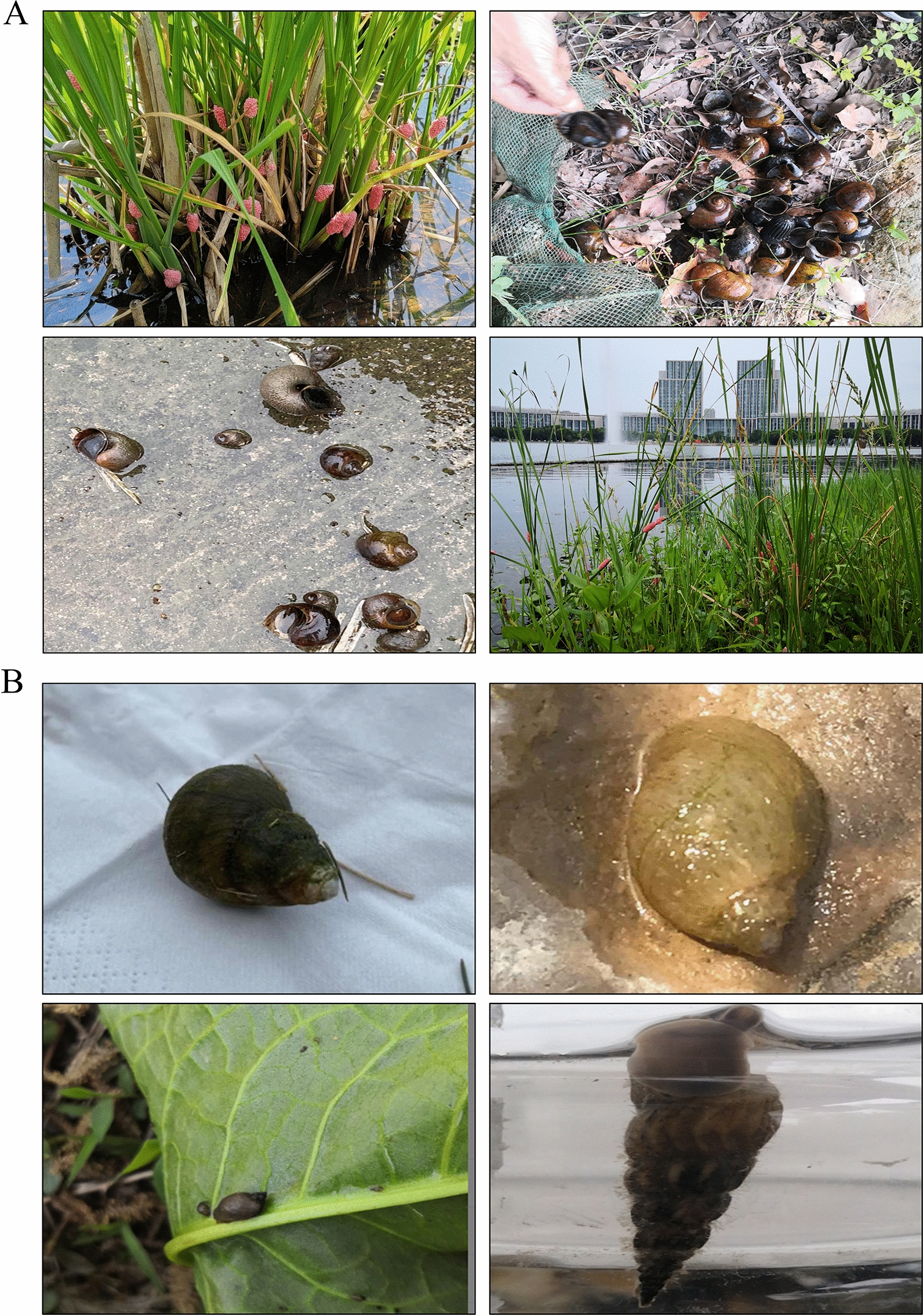
Photos reported through the ASI surveillance system. **A** Confirmed photos of apple snails. **B** Photos of other snails

We have thus far collected reported data on apple snails for 2 consecutive years (Additional file 1: Dataset S1). Figure 2A shows the confirmed photographs uploaded by different users, including those of adult snails and eggs. However, many of the reported photos (Fig. 2B) were mistaken identifications of no apple snails. In addition, there were also other phenomena such as not reporting on site or taking some online photos for reporting.

**Fig. 3:**
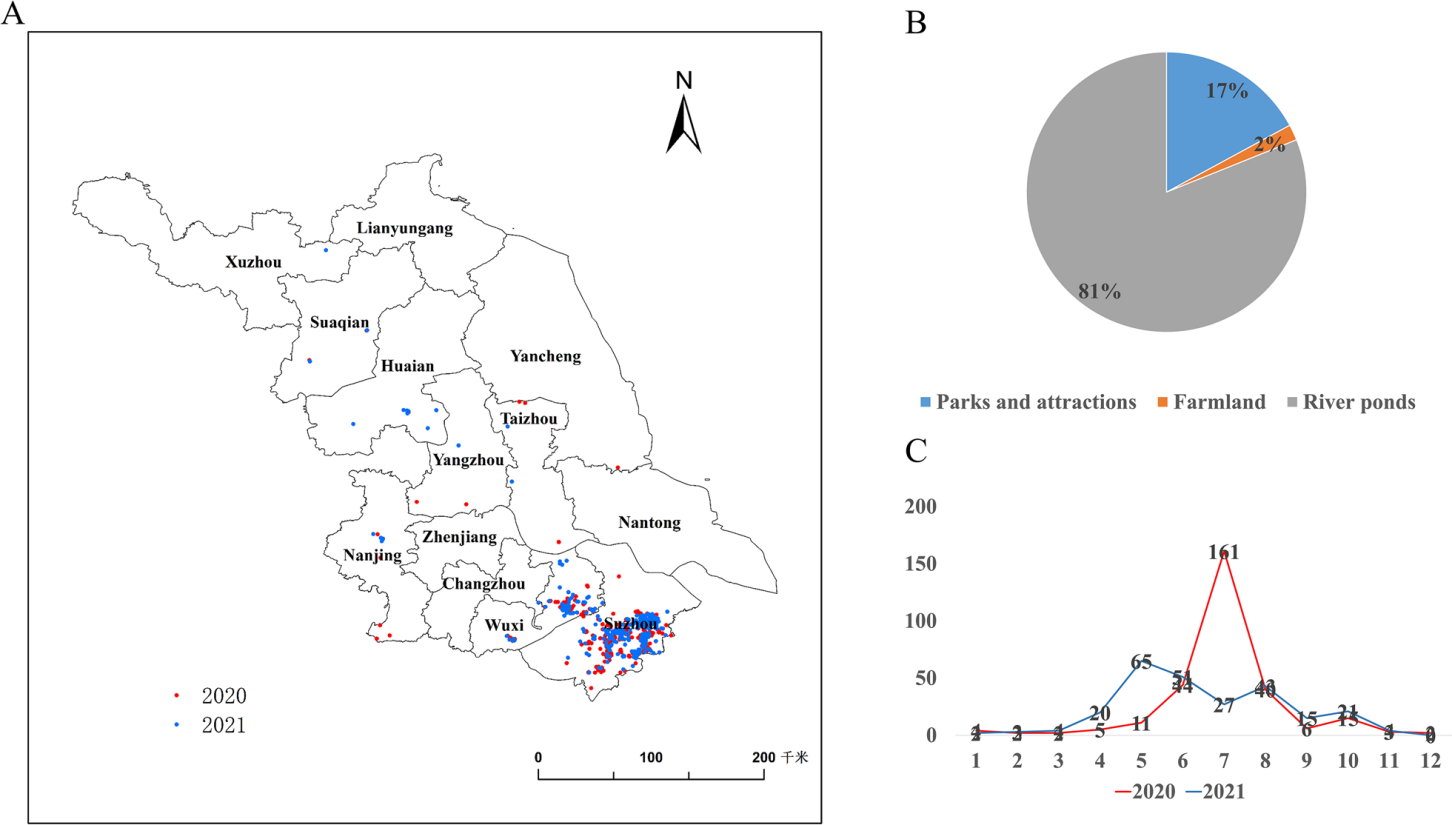
Two-year reporting data on apple snails through the ASI surveillance system. **A** Distribution of apple snails in the province in 2020 and 2021. **B** Ratios of apple snails invading various water environments. **C** Changes in the reported number of sightings of apple snails in different months in 2020 and 2021

By using the three criteria stated above, we identified 550 locations of apple snails in Jiangsu in 2020 and 2021 (Fig. 3A), covering almost the entire province. The largest number of sites were reported in Suzhou and Wuxi, 405 and 111, respectively, followed by Nanjing, Huaian, Taizhou, Suqian, Yangzhou, Changzhou, Xuzhou and Yancheng. No sites were reported in only three cities—Nantong, Lianyungang and Zhenjiang. Apple snails have destructive effects on different ecosystems. We found that they had successfully invaded ponds (81%), parks (17%) and farmland (2%), rendering them ecologically vulnerable (Fig. 3B). Most of the photos were those of snail eggs and were mainly captured in June, July and August of each year (Fig. 3C)*.*

## Discussions

The apple snail is a problematic invasive species in China. Inspired by the concept of mHealth, we developed the ASI system based on the WeChat platform to encourage the public to report breeding sites of the snail. This system can help us clearly understand the distribution of apple snails in Jiangsu Province of China and should provide breeding location information for its prevention and control in future.

The proposed system has several advantages over traditional methods for invasive species surveillance, including proactive surveillance [29], integrated interactive health education and public engagement in health programs [30, 31]. Apple snails have bright pink-red eggs that can easily be identified by the general public. We designed the proposed mobile surveillance system to monitor apple snails through public participation and real-time reporting. The results show that it is popular and effective. We plan to further publicize and maintain our system. We also reviewed the reported information and responded to users promptly to enhance their sense of participation in the enterprise. We have increased rewards for the correct identification of the species to motivate greater participation. A few incorrect results were also reported, which reflected poor public understanding of apple snails.

The distribution of apple snails through time indicates that the snails have gradually spread from the southeast coast to the middle and western parts of China and have a trend of northward diffusion probably influenced by human factors, water flow, global warming and other environmental factors [11]. In previous studies, apple snails were reported only in confined areas, and a detailed distribution of the species in Jiangsu Province has not been reported previously. Our results here yielded such a detailed distribution, and we identified 550 sites over 2 years covering nine cities. The sites were mainly concentrated in Suzhou and Wuxi. The dense water network in southern Jiangsu provides suitable natural conditions for the natural transmission and dispersal of apple snails. They might be transported through human activities, intentional or otherwise, as well as through natural dispersal based on geography, climate and water flow. The spread of apple snails from southern Jiangsu to central and northern parts of the province has increased significantly in recent years.

No sites were reported in three cities of the province. This might have occurred because of poor public awareness or inadequate promotion of the surveillance system in these areas. There was also a large gap in the number of reports between cities, possibly due to variations in the effectiveness of the promotion of the proposed system in these regions. We plan to further publicize our system with the help of local health departments. This could yield greater public participation, and more detailed information on the distribution of apple snails in the province, and potentially the entire country, may be gathered.

Apple snails devastate a wide range of crops, most notably rice seedlings. One adult snail can consume 5–24 rice seedlings per day [32]. An additional concern is the substantial increase in the global distribution of human infections with *A. cantonensis*, with cases reported in more than 30 countries. The global distribution of *A. cantonensis* infection coincides with the geographical distribution of their intermediate snail hosts [33]. Aggressive invasion by apple snails of new locations thus poses a significant threat to human health. We developed this system to monitor the spread of apple snails and to raise the attention of the government to strengthen control measures. In addition, the public will improve their awareness of apple snails by participating in our game, and this might lead to avoidance of *A. cantonensis* infection. The survival and reproduction of apple snails are dependent on the presence of water sources. However, the harm to wetland ecosystems caused by snail invasion may be underestimated and has not been adequately evaluated [34]. Our system can be used to determine whether apple snails have invaded wetlands, farmland and fresh water bodies and provides support for the early warning of its invasion. Most reports of the presence of apple snails occurred between May and September of each year, consistent with the peak spawning period of this species [35].

The study had several limitations. First, there was a sampling bias; at the initial stage of the study, our main purpose was to qualitatively understand whether snails breed in a particular area, but we did not measure the numbers of snails at each site. With the sustained running and reporting of the ASI system, this bias will be reduced in the future. At present, we have released the application primarily to our “circle of friends on WeChat.” Going forward, we will advertise the application through further wider channels, and we will also add drones to our surveillance system, targeting certain wild fields with few people. Second, we did not carry out a strict quantitative analysis in this study. The monitoring platform is only applied in Jiangsu Province at present, so we have only performed a simple internal test. Next, we plan to continue to optimize our platform and formulate usability metrics, so that the platform can be promoted and applied to a wider range. Lastly, the app was only available in Chinese, and it should also be available in other languages. This will allow the app to be distributed in many countries where apple snails are highly prevalent.

## Conclusions

The invasiveness of apple snails poses a significant challenge to public health in China. Our mobile health-based crowd-sourced system for the real-time reporting of this species of snails is helping identify their locations. This will strengthen the epidemiological capabilities of health authorities and empower the public to participate in active surveillance programs. From an academic standpoint, our work is a successful example of the use of mobile phones in epidemiology and health communication.

### Supplementary Information

**Additional file 1: Dataset S1.** Reported results of confirmed apple snails breeding places in 2020 and 2021.

**Additional file 2: Figure S1.** Promotional tweets on the official account of the proposed system during holidays. (A) May Day. (B) Mid-Autumn Festival. (C) The Dragon Boat Festival. (D) National Day.

## Data Availability

All data analyzed during this study are included in this article.

## Abbreviations

ASI: Apple snail inspector
GPS: Global positioning system
mHealth: Mobile health
*A. cantonensis*: *Angiostrongylus cantonensis*
